# A Recurrent Mucinous Neoplasm Originating From an Ovarian Mucinous Cystadenoma After an Adnexectomy As the First Procedure: A Case Report

**DOI:** 10.7759/cureus.31258

**Published:** 2022-11-08

**Authors:** Satoshi Ohira, Akiko Hayashi, Reika Kitano, Kyoko Tanaka, Fumiaki Kitamura

**Affiliations:** 1 Obstetrics and Gynecology, Marunouchi Hospital, Matsumoto, JPN

**Keywords:** mucinous borderline tumor, adnexectomy, ovary, mucinous cystadenoma, recurrent mucinous neoplasm

## Abstract

Ovarian mucinous cystadenomas are benign, but they can rarely recur if incompletely excised. We are the very first to report a case of recurrent mucinous neoplasm originating from an ovarian mucinous cystadenoma after adnexectomy as the first procedure. A 58-year-old woman was referred to our hospital with a two-year history of abdominal fullness. Magnetic resonance imaging (MRI) demonstrated a pelvi-abdominal cyst measuring 37 cm, without solid components within the cyst. A laparotomy revealed a huge cystic tumor originating from the right ovary. A right adnexectomy was performed without intraoperative cyst rupture or spillage. Histologically, the cyst was diagnosed as a mucinous cystadenoma. A month after the operation, ultrasonography revealed a cystic lesion measuring 1.8 cm adjacent to the right side of the uterine body. During the follow-up every three months, the cyst enlarged gradually, and an MRI performed 42 months after the operation revealed a cystic mass measuring 5.5 cm, including an internal protrusion. The second laparotomy revealed a cystic mass arising from the right surface of the uterine body, and a total hysterectomy and left adnexectomy were performed. Histologically, this uterine tumor was diagnosed as a mucinous borderline tumor that recurred from the ovarian mucinous cystadenoma. On histological examination of the resected uterus, the silken threads used at the first operation were observed in proximity to the tumor lesion. We speculated that the reason for the recurrence of our case may be the uterine-side remanence of the mucinous tumor cells from the first operation. Because the utero-ovarian ligament became short due to the large ovarian cyst, adnexectomy as a first procedure may be insufficient. A close follow-up of these patients is required for early detection of the recurrence, and attention is necessary for patients having malignant transformation due to an adenoma-borderline-malignant sequence of ovarian mucinous tumors.

## Introduction

Mucinous tumors are the second most common type of tumor of epithelial origin, comprising 8%-10% of ovarian tumors [1]. They are divided into benign, borderline, and malignant tumors. Mucinous cystadenomas are benign but can rarely recur if incompletely excised [2]. Risk factors for mucinous cyst recurrence are cystectomy as the first procedure and the occurrence of intraoperative cyst rupture and spillage [3]. Herein, we report a rare case of recurrent mucinous neoplasm originating from an ovarian mucinous cystadenoma after adnexectomy as the first procedure. Moreover, the recurrent tumor exhibited the features of a mucinous borderline tumor arising from the surface of the uterine body.

## Case presentation

**Figure 1:**
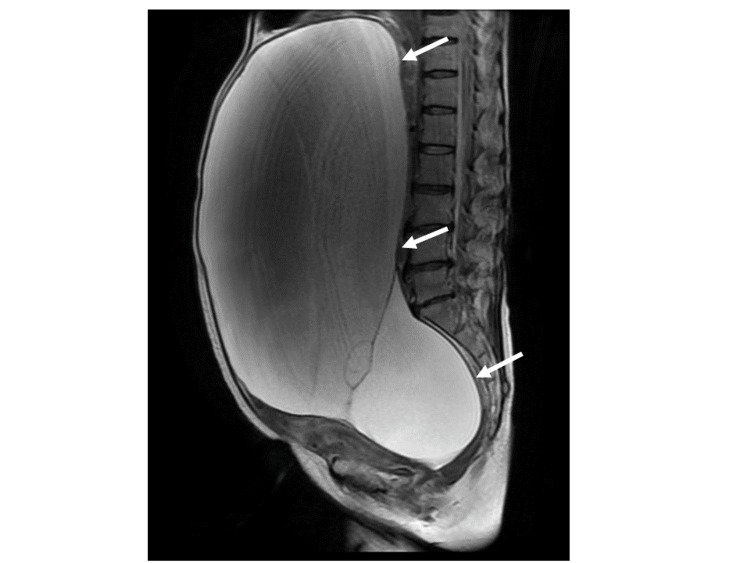
Ovarian cyst MRI (T2-weighted imaging) demonstrates a huge pelviabdominal cyst measuring 37 cm without solid components within the cyst (arrows).

A 58-year-old postmenopausal Japanese woman (gravida 3, para 2, miscarriage 1) was referred to our hospital with a two-year history of abdominal fullness. The patient was vitally stable, and the ultrasound revealed a large pelvi-abdominal mass with internal echoes, single septa, and no ascites. With the simple ultrasound-based rules of International Ovarian Tumor Analysis (IOTA) [4], the cystic tumor was classified as benign. She had undergone an appendectomy for appendicitis at 10 years old. Serum carbohydrate antigen 19-9 (CA19-9) level and cancer antigen 125 (CA125) level were high at 224.2 U/mL and 223.1 U/mL, respectively. Serum carcinoembryonic antigen (CEA) level was slightly high at 7.2 ng/mL. MRI demonstrated a large cystic tumor measuring 37 x 17 x 29 cm. The atrophic uterus was being pushed out by the large tumor. Both T1- and T2-weighted imaging revealed no solid components within the cyst (Figure 1). An ovarian cyst was suspected, and surgical intervention was planned.

 

**Figure 2:**
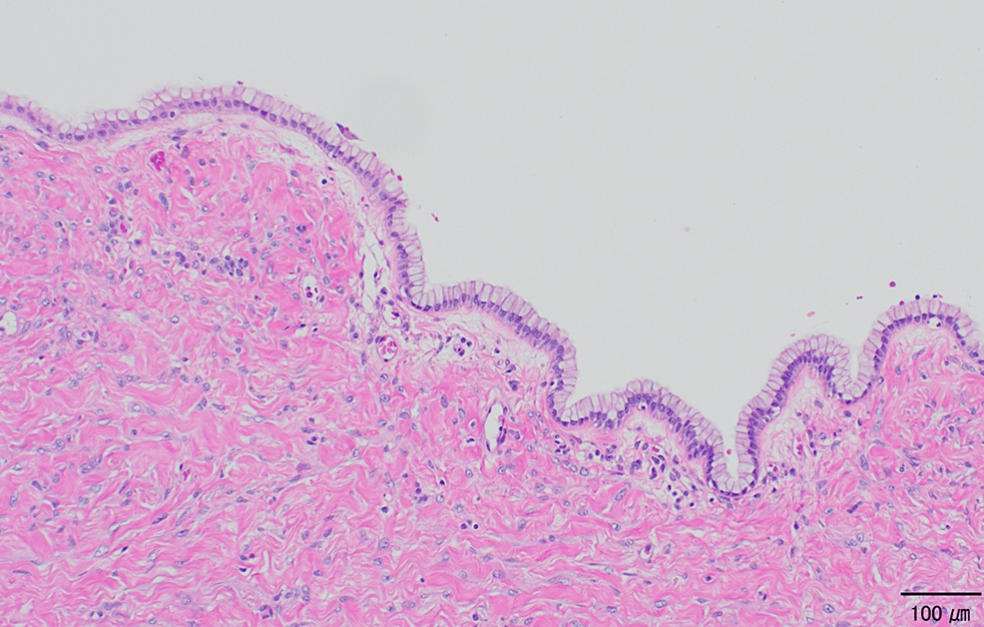
Mucinous cystadenoma Histological findings of the resected right adnexa at the first operation (hematoxylin and eosin staining). The cyst wall is lined by simple, non-stratified mucinous epithelium without atypia.

A laparotomy with a vertical incision revealed a huge cystic tumor originating from the right ovary. There was no adhesion in the abdominal cavity. The cyst fluid was aspirated by a SAND balloon catheter [5] without intraperitoneal spillage. The cyst collapsed gradually, and nine liters of liquid, light yellow in color, were aspirated. The left ovary and uterus were unremarkable, and a right adnexectomy was performed. Intraoperative ascitic cytology was negative. On histological examination, the cyst wall was lined by simple non-stratified mucinous epithelium without atypia (Figure 2). The tissue specimen was made for every 1 cm of the cyst, and the atypical cell was not detected in any specimen. We diagnosed this case as a mucinous cystadenoma of the right ovary.

**Figure 3:**
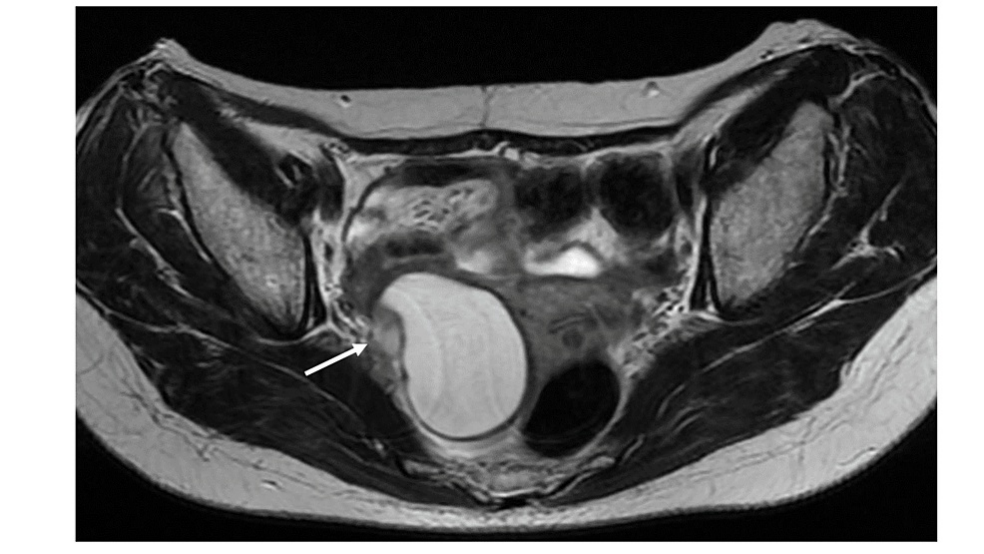
Recurrent tumor An MRI (T2-weighted imaging) performed 42 months after the operation reveals a cystic mass measuring 5.5 cm on the right side of the uterus. The cyst includes an internal protrusion (arrow).

One month after the operation, ultrasonography revealed a cystic lesion measuring 1.8 cm adjacent to the right side of the uterine body. The cyst had no solid component. As the position of the cyst was on the ipsilateral side of the adnexectomy, we considered that it was a peritoneal inclusion cyst. During the follow-up every three months, the cyst enlarged gradually, and an MRI performed 42 months after the operation revealed a cystic mass measuring 5.5 cm on the right side of the uterus. As the cyst included an internal protrusion on the MRI, we considered it a neoplastic tumor (Figure 3). With the simple ultrasound-based rules of IOTA [4], the cyst could not be classified. Serum CA19-9 level and CEA level were high at 76.5 U/mL and 7.6 ng/mL, respectively, whereas the CA125 level was normal.

**Figure 4:**
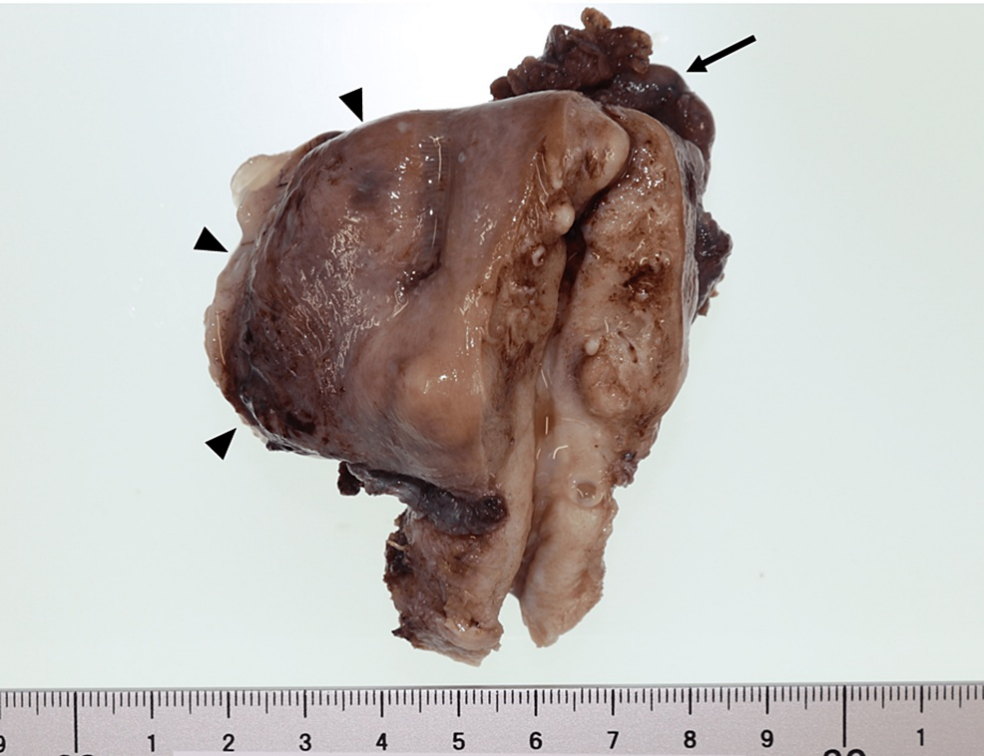
Uterine cyst Macroscopically, the resected uterus has a cystic mass arising from the right surface of the uterine body (arrowheads). Arrow; left adnexa.

**Figure 5:**
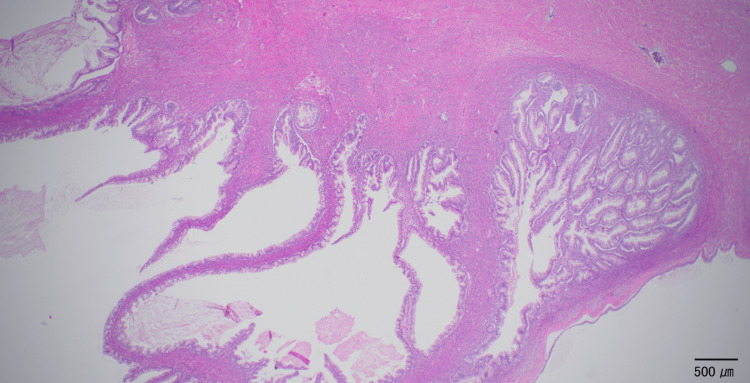
Mucinous borderline tumor Histological findings of the uterine cyst (hematoxylin and eosin staining). A part of the cyst wall is lined by stratified, proliferative mucinous epithelium exhibiting tufted and papillary intraglandular growth.

Forty-six months after the first operation, a second laparotomy was planned to confirm the diagnosis. A macroscopic examination of the surgical procedure revealed a cystic mass arising from the right surface of the uterine body, and a total hysterectomy and left adnexectomy were performed. Intraoperative ascitic cytology was negative. Macroscopically, the uterine cyst measured 5 x 3 cm and contained light-yellow mucus, and the left adnexa was unremarkable (Figure 4). On histological examination, the cyst wall was lined dominantly by simple, non-stratified mucinous epithelium, but part of it was lined by stratified, proliferative mucinous epithelium exhibiting tufted and papillary intraglandular growth; moreover, goblet cells were present (Figure 5). The mucinous epithelium displayed mild to moderate nuclear atypia and some mitotic activity. There was significant proliferation; however, there was no evidence of a destructive infiltrative growth pattern. We diagnosed this uterine tumor as a mucinous borderline tumor that recurred from the ovarian mucinous cystadenoma. A month after the second operation, we performed a restaging laparotomy with peritoneal washing cytology, an omentectomy, and a biopsy of the peritoneum. No evidence of tumor cells was observed microscopically. The patient was alive and well 60 months after the first operation.

## Discussion

The recurrence of ovarian mucinous cysts is rare. Nine recurrent cases originating from benign mucinous cystadenomas of the ovary have been reported [1, 2, 6-12]. In all these cases, a cystectomy had been performed as the first procedure. In addition, Ben-Ami et al. studied 42 cases of women who underwent the removal of benign mucinous adnexal cysts by either adnexectomy or cystectomy [3]. Three of the 42 women had mucinous cyst recurrence in the ipsilateral ovary, and all three underwent cystectomy in the first surgical procedure in which there was intraoperative cyst rupture and spillage [3]. Therefore, our case is the first reported case of a recurrent mucinous tumor of the ovary after adnexectomy as the first procedure.

Among the above nine cases of recurrent mucinous tumor of the ovary, eight cases had a recurrence in the ipsilateral ovary as a mucinous cystadenoma histologically [1, 6-12]. Another case had recurrence as a mucinous cyst within a cesarean scar of the myometrium, and the recurrent cyst was a mucinous cystadenoma with focal borderline changes histologically [2]. Therefore, our case is the second case report of a mucinous ovarian neoplasm that recurred within the myometrium as a mucinous borderline tumor.

In this case, although an adnexectomy was performed without intraoperative rupture and spillage, the ovarian mucinous cystadenoma recurred on the ipsilateral side of the uterine surface. On histological examination of the resected uterus, the silken threads used at the first operation were observed in proximity to the tumor lesion. Therefore, we speculate that the reason for the recurrence may be the uterine side remanence of the mucinous tumor cells from the first operation. Because the utero-ovarian ligament became short due to the large ovarian cyst, the adnexectomy may have been insufficient microscopically. Although the recurrent uterine tumor may be a benign mucinous cyst at the beginning of the recurrence, a mucinous borderline component may arise due to an adenoma-borderline sequence of ovarian mucinous tumors [13].

## Conclusions

We report the first case of a recurrent benign mucinous tumor of the ovary after adnexectomy as the first procedure. In the case of a large mucinous ovarian cyst, even though an adnexectomy was performed, tumor cells may remain on the uterine side due to the shortened utero-ovarian ligament. A close follow-up of these patients is required for early detection of recurrence, and attention is necessary for malignant transformation due to the adenoma-borderline-malignant sequence of ovarian mucinous tumors.
